# Variation in Seed Dormancy of Chaco Seasonally Dry Forest Species: Effects of Seed Traits and Population Environmental Conditions

**DOI:** 10.3390/plants12091790

**Published:** 2023-04-27

**Authors:** Tania Bertuzzi, Diego López-Spahr, Carlos A. Gómez, Silvia Sühring, Gisela Malagrina, Carol C. Baskin, Guadalupe Galíndez

**Affiliations:** 1 Centro de Investigaciones y Transferencia de Catamarca (CITCA)—CONICET, Facultad de Ciencias Agrarias, Universidad Nacional de Catamarca, Catamarca 4700, Argentina; taniabertuzzi13@gmail.com; 2 Facultad de Ciencias Naturales, Universidad Nacional de Salta, Salta 4400, Argentina; 3 Laboratorio de Microscopía Electrónica de Barrido (LASEM)—CCT-CONICET, Universidad Nacional de Salta, Salta 4400, Argentina; 4 Banco Base de Germoplasma, Instituto de Recursos Biológicos, CIRN-INTA, Hurlingham, Buenos Aires 1686, Argentina; 5 Department of Biology, University of Kentucky, Lexington, KY 40506-0225, USA; 6 Department of Plant and Soil Sciences, University of Kentucky, Lexington, KY 40546-0321, USA; 7 Facultad de Ciencias Naturales, Universidad Nacional de Salta—CCT-CONICET, Salta 4400, Argentina

**Keywords:** Anacardiaceae, arid environments, Fabaceae, interspecific variability, intraspecific variability, physical dormancy, physiological dormancy, Rhamnaceae, seed traits, water gap

## Abstract

The persistence of subtropical seasonally dry forests urgently requires the implementation of ex situ conservation and restoration programs. We studied variation in seed traits and dormancy of six native species growing in seasonally dry Chaco forests of Argentina. We documented high intra- and interspecific variability in seed traits and dormancy. Fresh seeds of *Geoffroea decorticans* and *Parasenegalia visco* (Fabaceae) were water-permeable and nondormant (ND), while those of *Parkinsonia praecox* and *Vachellia aroma* (Fabaceae) were water-impermeable and had physical dormancy (PY). Seeds of *Schnopsis lorentzii* (Anacardiaceae) and *Sarcomphalus mistol* (Rhamnaceae) were water-permeable and had physiological dormancy (PD). Mechanical and chemical scarification were the most effective methods to break PY, and dry storage for 3 months was effective in breaking PD. Seeds of large-seeded species were ND or had PD, and those of small-seeded species had PY. Species inhabiting moist habitats had ND seeds, whereas those from seasonally dry habitats had seeds with PY or PD. These results suggest that seed traits and dormancy are species-specific and that intraspecific variation in seed traits is likely associated with high phenotypic plasticity of species in response to local environmental heterogeneity. These findings should be considered at the time of implementation of conservation techniques and for seed sourcing decisions for restoration.

## 1. Introduction

The subtropical seasonally dry forests of Argentina, like many other forests in the world, have suffered great destruction due to deforestation, overgrazing, fires, and land-use changes to agriculture [1]. In addition, numerous native species have high value for use as wood sources and are being overexploited for charcoal, building materials, and/or for medicinal and/or ornamental purposes. This situation has led to the impoverishment and reduction of native forests [2], and many species have become locally extinct or are threatened regionally, making ex situ conservation and ecological restoration a priority for these forests. The storage of seeds in gene banks is the most practicable and least expensive way to conserve plant biodiversity [3]. For successful seed storage to ensure current and/or future uses, it is necessary to determine seed storage behavior and develop standard protocols for seed dormancy-break and germination [4]. Moreover, morphological/physical (e.g., seed mass, seed moisture content, seed coat features) and physiological (e.g., seed dormancy) seed traits are in many cases a prerequisite for management decisions and successful ecological restoration [5,6]. In this sense, Seglias et al. [7] highlighted the relevance of identifying intraspecific variation in seed dormancy and germination before recommending the use of specific seed sources and whether restoration can be accomplished at the species level.

Seed dormancy is an important adaptive trait that helps to ensure that seeds germinate only when conditions are favorable for successful seedling establishment and growth [8]. Among the classes of seed dormancy, physiological dormancy (PD) is the most common and occurs in different plant lineages and ecosystems around the world [9]. PD is due to a physiological inhibiting mechanism in the embryo (e.g., high level of abscisic acid in the embryo) that decreases the growth potential of the embryo, thus preventing it from being able to overcome the mechanical resistance of the endosperm, perisperm, seed coats or an indehiscent fruit wall [10]. The second most common class of dormancy is physical dormancy (PY), which is caused by the presence of water-impermeable palisade cell layer(s) in the seed or fruit coat; it has been reported in 18 angiosperm families, including Anacardiaceae, Fabaceae, and Rhamnaceae [8]. Although PY has been linked to phylogeny, there are species in these families whose seeds are nondormant (ND) or have PD or PY+PD [8].

Species with PY also have been documented in different ecosystems around the world, although they are more prevalent in dry ecosystems with seasonal rainfall, relatively high temperatures and low relative humidity than in moist environments with aseasonal rainfall, relatively low temperatures, and high humidity [9,11,12]. Baskin and Baskin [13] proposed that the selective force for evolution of PY was climatic drying, resulting in seasonally dry forest habitats within the tropics and subtropics. Thus, PY evolved as a mechanism for delaying germination until the beginning of next rainy season in seasonally dry environments. These authors also mentioned that large-seeded species with a high water content at maturity and intolerance to desiccation (i.e., recalcitrant species) are not compatible with seasonally dry environments. On the other hand, small seeded species with a low water content and tolerance to desiccation (i.e., orthodox seeds) are characteristic of these environments and have PY or PD. In this sense, Rubio de Casas et al. [12], taking Fabaceae as a case study, proposed that within a given lineage, taxa producing small dormant seeds will predominate in seasonal environments, whereas large ND seeds will predominate in aseasonal environments. Thus, PY gives small seeds an adaptive advantage by delaying seed germination until the dry season ends and the moist season begins, which would be the most favorable time for seedling establishment.

Seeds with PY have a morpho-anatomical specialized region, called a ‘water-gap’, that differs anatomically from the rest of the seed coat [14]. Species of Anacardiaceae with PY have a water-impermeable fruit coat, and the water-gap is a specialized structure in the endocarp (carpellary micropyle) [15]. Seeds of Fabaceae and Rhamnaceae with PY have a water-impermeable seed coat, and the water gap is a lens and hilar slit, respectively [8,14]. The water gap acts as an environmental signal detector, and it opens in response to specific conditions thereby allowing water to enter the seed thus promoting germination [14]. The location, morpho-anatomy, and origin of water gaps can vary between and even within families [8,14]. In natural environments, seasonal temperature variation, high or low temperatures, and/or fires are signals that can disrupt or dislodge the water gap marking the termination of dormancy. However, in laboratory mechanical and chemical scarification, dry or wet heat conditions and even dry storage can break PY or make seeds sensitive to dormancy-breaking treatments [8,16,17,18]. In addition, Rodrigues-Junior [19] reported that large seeds of *Senna multijuga* (Fabaceae) are more sensitive to dormancy break than small seed due to their high MC, relatively thin seed coat, and low seed coat thickness: seed mass ratio. After seeds or fruits become permeable to water, they can germinate in both light and darkness over a wide range of temperatures [13].

In addition, several studies have reported within-species variation in seed traits, seed dormancy and germination that is associated with climatic differences among sites and/or with different environmental conditions during seed maturation (i.e., maternal environmental effect) [8,9,20]. For example, Rubio de Cases et al. [12] and Renzi et al. [21] documented a positive association between seed dormancy and latitude, while Fernández-Pascual et al. [22] reported a positive correlation between dormancy and altitude and with sites with high temperature variation. Further, the development of the water-impermeable coat occurs at the final phase of seed maturation when the seed MC decreases to 8–15%, depending on the species and the maternal environmental conditions during this phase [23]. Thus, depending on the maternal environmental factors, the structure and thickness of the seed coat can vary among populations and over the years [18]. Plant species exhibit a high ability to generate different phenotypes in response to variation in the environment (i.e., phenotypic plasticity) [21].

The Chaco subtropical dry forest in northwestern Argentina is an example of a bioregion with a seasonal environment [24,25]. Precipitation is erratic with marked differences between the wet (predominantly summer) and dry (with marked water deficit) seasons, and temperatures tend to be high (≥30 °C on average in summer) but vary with latitude and altitude [25]. Three vegetation units can be distinguished in the Chaco forests based on elevation and amount of annual precipitation: (1) Arid Chaco in the Central Valley of Catamarca Province at 200 and 600 m a.s.l. receives 350–650 mm of rain, (2) Semiarid Chaco in the eastern part of Catamarca Province at 300 and 600 m a.s.l. receives 650–1000 mm of rain, and (3) Chaco Serrano on mountain extensions at 600 and 1500 m a.s.l. receives 500–800 mm of rain [25,26]. Some of the characteristic native trees of the Chaco forest include the Anacardiaceae species *Schinopsis lorentzii;* the Fabaceae species *Geoffroea decorticans*, *Parasenegalia visco*, *Parkinsonia praecox,* and *Vachellia aroma*; and the Rhamnaceae species *Sarcomphalus mistol*. *P. visco* and *V. aroma* are characteristic species of Chaco Serrano; *S. mistol*, *P. praecox*, and *G. decorticans* of Arid and Semiarid Chaco; and *S. lorentzii* of Chaco Serrano and Semiarid Chaco.

Previous studies concluded that seeds of *G. decorticans* [15], *P. praecox* [27,28,29,30], *S. mistol* [27,31], *S. lorentzii* [32], and *V. aroma* [32,33,34] have PY. It also has been reported that seeds of *G. decorticans* [17,35] and *S. mistol* [36] are ND, and species of *Schinopsis* [37] are reported to have PD or be ND. There is no information about dormancy of *P. visco* seeds. In this context, knowledge of seed traits, type of seed dormancy, seed dormancy-breaking mechanism, and the relation between seed dormancy and the environmental conditions in the habitat of each species is key for predicting plant regeneration and population dynamics of the species in natural and anthropized environments. Additionally, this information is required for efficient use of seeds in restoration and conservation programs, both of which are urgently required for the persistence of the Chaco seasonally dry forests in Argentina (National Native Forests Law N° 26.331/2007).

The broad objective of our research was to study seed dormancy of these six Chaco species, evaluating their inter- and intraspecific variability in relation to seed traits and environmental variables. All these species can grow in each of the three kinds of Chaco forests, but due to differences in environmental conditions in the forests it is possible that seed dormancy and seed traits differ among species and/or populations [38]. Our first hypothesis was that Anacardiaceae, Fabaceae species (except for *G. decorticans* that has ND seeds [17]), and Rhamnaceae have seed dormancy (PY and/or PD). We expected seeds of *G. decorticans* to have a permeable seed coat and those of the other species to have a water-impermeable coat (PY) and/or permeable coats but low or no initial germination (PD). Our second hypothesis was that seed dormancy is related to seed traits. We expected that small-seeded species and/or populations have seeds with a relatively low moisture content (MC), a thick seed coat, and therefore PY; whereas large-seeded species and/or populations have seeds with a relatively high MC, a thin seed coat, and therefore ND or PD. Finally, our third hypothesis was that seeds of species and/or populations occupying the same environment (i.e., vegetation units) have the same class of dormancy. We expected species and/or populations from Arid Chaco to have dormant seeds (PY or PD) and those from Semiarid and Serrano Chaco to have ND seeds.

## 2. Results

### 2.1. Seed Traits

**Figure 1:**
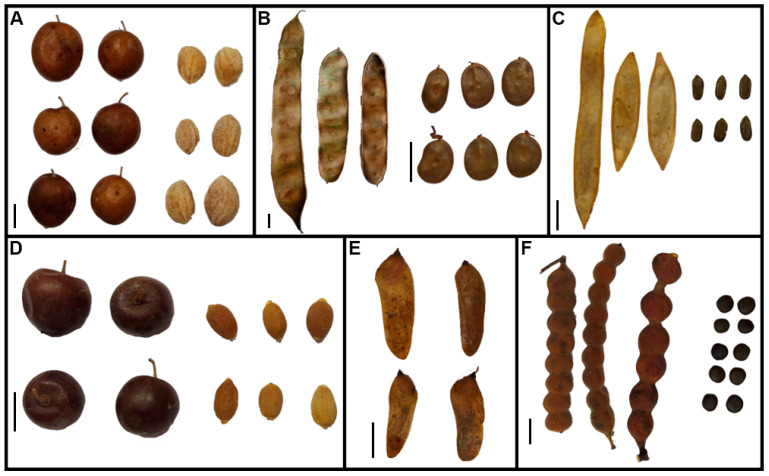
Fruit and seed morphology of *Geoffroea decorticans* (**A**), *Parasenegalia visco* (**B**), *Parkinsonia praecox* (**C**), *Sarcomphalus mistol* (**D**), *Schinopsis lorentzii* (**E**), and *Vachellia aroma* (**F**). Bars = 10 mm.

Initial seed viability was on average ≥ 80% for all species, with no significant differences among populations (*p* > 0.05). MC and seed mass varied among species and, depending on the species, between populations (Table 1). Seeds of *P. visco*, *P. praecox*, *V. aroma*, and *S. lorentzii* had on average a higher MC (8.9%) than those of *S. mistol* (7.9%) and *G. decorticans* (5.8%). For *P*. *visco*, *S. mistol*, and *V. aroma*, differences in seed MC between populations were observed. Seed mass ranged from <50 mg in *P. praecox* to >700 mg in *G. decorticans* (Table 1 and Figure 1). Except for *P. praecox* and *S. lorentzii*, seed mass varied among populations of a species. Seeds of *P. visco* germinated to almost 100% in all light and temperature conditions, and the time to 50% germination (t_50_) was low and similar among populations and treatments (>3 d). Germination of *G. decorticans* was higher in darkness at 25 °C (≥78%) than at the other conditions, with no differences among populations. For the other species, germination was lower than 50% and differed between populations and/or treatments, depending on the species (Table 1).

**Figure 2:**
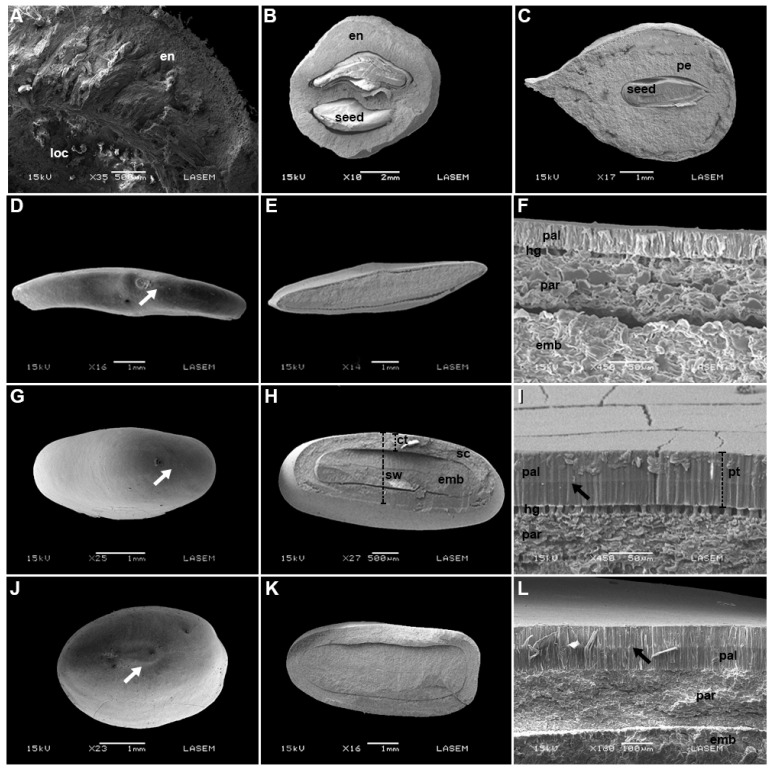
Scanning electron micrographs of cross sections of the fruit of *Geoffroea decorticans* (**A**), *Sarcomphalus mistol* (**B**), and *Schinopsis lorentzii* (**C**). Scanning electron micrographs of water gap region of seeds of *Parasenegalia visco*: (**D**) hilar–micropilar region, (**E**) cross section, (**F**) detail of seed coat; *Parkinsonia praecox*: (**G**) hilar–micropilar, (**H**) cross section, (**I**) detail of seed coat; *Vachellia aroma*: (**J**) hilar–micropilar, (**K**) cross section, (**L**) detail of seed coat. Abbreviations: emb, embryo; en; endocarp; hg, hourglass cells; loc, locule; pal, palisade layer; par, sclerified parenchyma; pe, pericarp; sc: seed coat. Black arrows: light-line, white arrows: lens. Bars with dotted lines represent measured traits: ct, coat thickness; pt, palisade layer thickness; sw, seed width.

The dispersal/germination unit for *G. decorticans*, *S. mistol*, and *S. lorentzii* is a fruit, and no water gaps were observed. *G. decorticans* and *S. mistol* have a woody endocarp composed of several layers of sclerified cells (Figure 1 and Figure 2A,B). *S. lorentzii* has a woody pericarp consisting of an exocarp with various layers of thick lignified cells, a mesocarp with an external layer of suberized cells and various layers of sclereids, and an endocarp with two or three layers of sclereids, that were smaller than those of the mesocarp (Figure 1 and Figure 2C). Seeds of *P. visco*, *P. praecox*, and *V. aroma* have a lens in the hilar–micropylar region (Figure 2D,G,J). The seed coat of *P. visco* has three zones: one macrosclereid layer (palisade) with no distinguishable light-line, one osteosclereid layer (hourglass cells), and various layers of sclerified parenchyma (Figure 2E,F). *P. praecox* has a seed coat also composed of three zones: one palisade layer with a light-line in the middle of the cells, one hourglass cell layer, and various layers of sclerified parenchyma (Figure 2H,I). *V. aroma* has a seed coat with two zones: one palisade layer with a light-line in the external half of the cells, and various layers of sclerified parenchyma (Figure 2K,L).

For seeds of *P. visco*, *P. praecox*, and *V. aroma*, thickness of palisade layer in the seed coat, seed coat thickness, and seed width varied among species and populations (Table 2). Seeds of *V. aroma* and *P. praecox* had the thickest palisade layer (>0.06 mm and ≤0.04 mm, respectively). Thickness of the palisade layer varied among populations of *V. aroma* but not *P. praecox*. *G. decorticans* had the thickest coat and widest seeds (>2.5 mm and 10 mm, respectively), whereas *P. visco* had the thinnest coat (≤0.14 mm) and *P. praecox* the narrowest seeds (≤2 mm). Only for *G. decorticans* did coat thickness vary among populations. The seed coat thickness: seed width and seed coat thickness: seed mass ratios varied among species, with *S. lorentzii* showing the highest ratios (≥0.40 and ≥0.010, respectively), and *P. visco* the lowest (≤0.12 and 0.001, respectively). Except for *S. lorentzii* and *V. aroma*, population differences were found for the seed coat thickness: seed width ratio.

### 2.2. Seed Dormancy

#### 2.2.1. Imbibition Curve

After a week of imbibition, intact and mechanically scarified seeds of *G. decorticans*, *P. visco*, *S. mistol* and *S. lorentzii* did not differ in percentage of water imbibed (Table 1). In contrast, the mass of mechanically scarified seeds of *P. praecox* and *V. aroma* increased at least 120%, whereas that of intact seeds only increased 60%. Significant differences in mass increase among populations occurred only for *V. aroma*.

#### 2.2.2. Dormancy-Breaking Treatments

**Figure 3:**
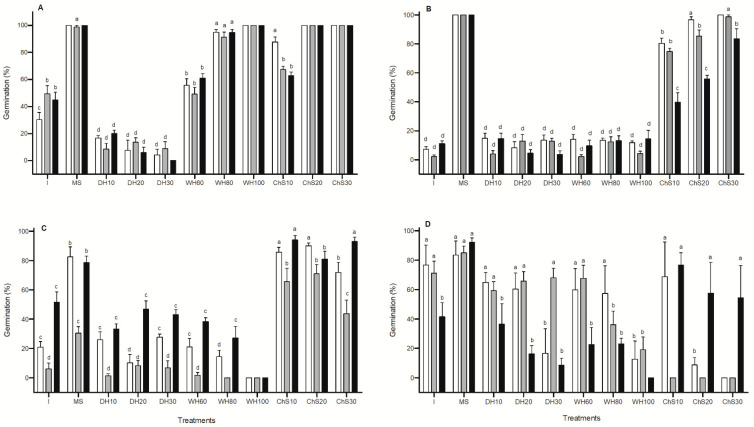
Seed germination (%) of (**A**) *Parkinsonia praecox*: Pomancillo Oeste (white bars), Recreo (grey bars), and Santa Cruz (black bars); (**B**) *Vachellia aroma*: Capital (white bars), Colonia del Valle (grey bars), and Pomancillo Oeste (black bars); (**C**) *Sarcomphalus mistol*: Huaycama (white bars), Pomancillo Oeste (grey bars), and Recreo (black bars); and (**D**) *Schinopsis lorentzii*: El Portezuelo (white bars), La Quebrada (grey bars), and Santo Domingo (black bars). Treatments: mechanical scarification (MS), dry heat (seeds placed in oven at 90 °C for 10, 20, and 30 min; DH10, DH20, DH30), wet heat (seeds immersed in water for 2 min at 60, 80, and 100 °C; WH60, WH80, WH100), chemical scarification (seeds immersed in sulfuric acid for 10, 20, and 30 min; Ch10, Ch20, Ch30) and intact seeds (I). Values with different letters indicate significant differences between populations and treatments included in the analyses (DGC test *p* < 0.05). Data are the means of 4 replicates (+1 standard error).

Germination percentages and t_50_ differed significantly among populations and treatments (Figure 3) for *P. praecox*, *V. aroma*, *S. mistol*, and *S. lorentzii*. For all populations of *P. praecox*, 95–100% germination occurred between 3 and 5 days after mechanical scarification, wet heat treatment (100 °C), and chemical scarification (20 and 30 min; Figure 3A). *V. aroma* seeds from all populations germinated to 100% in <2 days after mechanical scarification and those from Capital also after chemical scarification (30 min; Figure 3B). Three months of dry storage affected seed germination of *S. mistol* and *S. lorentzii* (Figure 3C,D). For *S. mistol*, storage increased germination of intact seeds from Recreo (from 28% to 52%), decreased germination of seeds from Huaycama (from 48% to 22%) and had no effect on germination of seeds from Pomancillo Oeste (3% and 6%; Table 1 and Figure 3C). Mechanical and chemical scarification increased germination, mainly for seeds from Huaycama and Recreo, and no germination was registered after wet heat treatment (100 °C) for all populations (Figure 3C). For *S. lorentzii*, storage significantly increased germination of intact seeds from El Portezuelo and La Quebrada (Table 1, Figure 3D). The response to different scarification and heat treatments varied depending on the population and treatments (Figure 3D).

**Figure 4:**
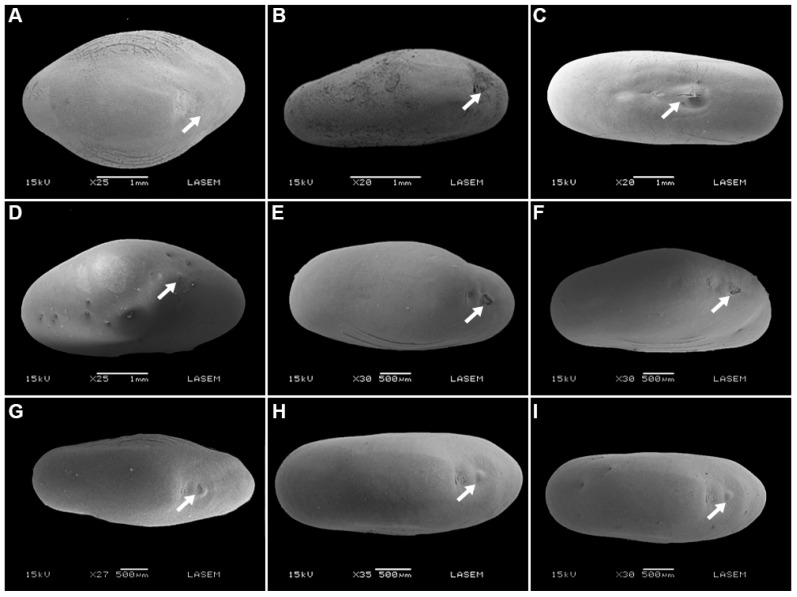
Scanning electron micrographs of water gap region of seeds after dormancy-breaking treatments for *Parkinsonia praecox*: (**A**–**C**): chemical scarification (seeds immersed in sulfuric acid for 10, 20, and 30 min, respectively); (**D**–**F**): wet heat (seeds immersed in water for 2 min at 60, 80, and 100 °C, respectively); (**G**–**I**): dry heat (seeds placed in oven at 90° for 10, 20, and 30 min, respectively). White arrows indicate the lens (water gap).

**Figure 5:**
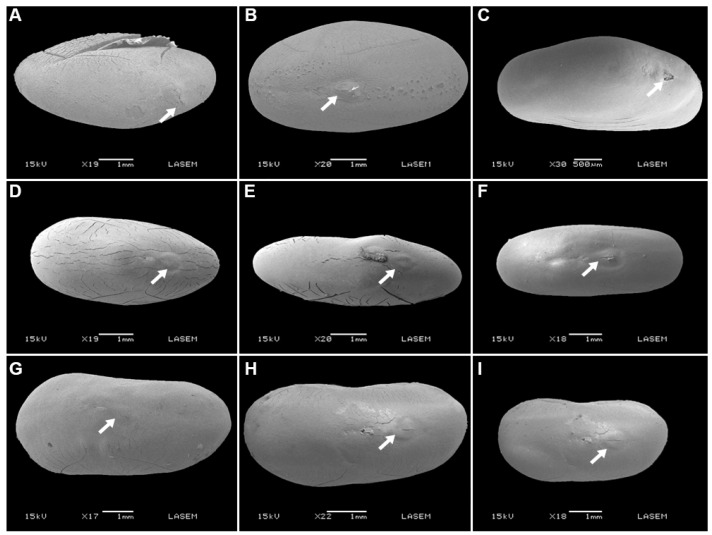
Scanning electron micrographs of water gap region of seeds after dormancy-breaking treatments for *Vachellia aroma*: (**A**–**C**): chemical scarification (seeds immersed in sulfuric acid for 10, 20, and 30 min, respectively); (**D**–**F**): wet heat (seeds immersed in water for 2 min at 60, 80, and 100 °C, respectively); (**G**–**I**): dry heat (seeds placed in oven at 90° for 10, 20, and 30 min, respectively). White arrows indicate the lens (water gap).

After chemical scarification, the water gap on seeds of *P. praecox* and *V. aroma* opened by removal or fissure of the lens (Figure 4 and Figure 5), and wet heat treatments but not dry heat treatments caused the lens on *P. praecox* seeds to open (Figure 4E,F).

### 2.3. Associations between MC, Seed Mass, Morpho-Anatomical Traits, and Geographic and Environmental Variables

**Figure 6:**
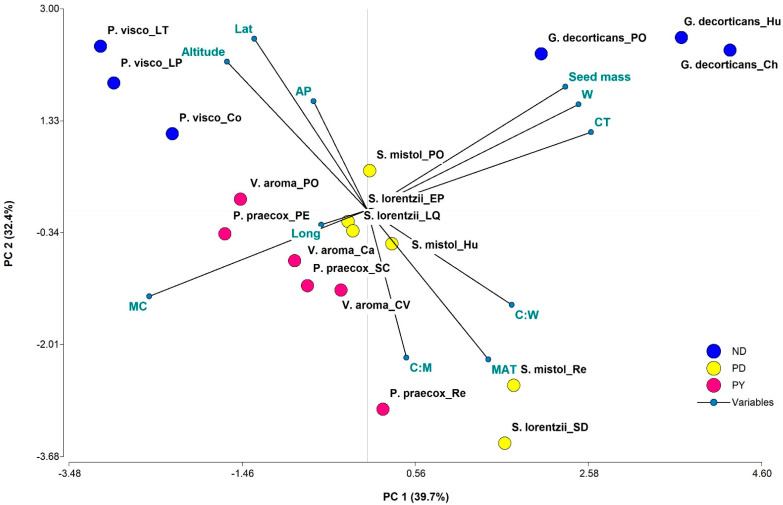
Ordination of species and populations on the first two principal components of the principal component analysis (PCA) based on seed traits (MC, seed mass, W, CT, C:W, C:M), environmental variables (latitude, longitude, altitude, MAT, AP) and seed dormancy (ND: blue, PY: magenta, PD: yellow). Abbreviations: Ch: Chacabuco, Hu: Huaycama, PO: Pomancillo Oeste, Co: Concepción, LP: La Puerta, LT: Los Túneles, PE: Pomenacillo Este, Re: Recreo, SC: Santa Cruz, EP: El Portezuelo, LQ: La Quebrada, SD: Santo Domingo, Ca: Capital, and CV: Colonia del Valle.

Seed MC correlated negatively with coat thickness (*r* = −0.92, *p* < 0.001), seed width (*r* = −0.95, *p* < 0.001) and seed mass (*r* = −0.95, *p* < 0.001), whereas coat thickness correlated positively with seed width (*r* = 0.96, *p* < 0.001) and seed mass (*r* = 0.93, *p* < 0.001). Seed width correlated positively with seed mass (*r* = 0.98, *p* < 0.001). The seed coat thickness: seed width ratio correlated positively with the seed coat thickness: seed mass ratio (*r* = 0.72, *p* < 0.001). Seed coat thickness: seed width and seed coat thickness: seed mass ratios correlated negatively with latitude (*r* = −0.57 and −0.56, respectively, *p* ≤ 0.02). Principal component analysis (PCA) showed that the first and second components accounted for 72.1% of total variation between species and populations for all variables considered in the analysis (Figure 6). PC1 explained 39.7% of the variation and ordered species and/or populations according to seed mass, seed coat thickness, and seed width. Thus, all *G. decorticans* populations, *S. mistol* (Recreo), and *S. lorentzii* (Santo Domingo) were located to the right of axis 1 and had high seed mass, seed coat thickness, and seed width, and low MC. In contrast, all populations of *P. visco, V. aroma*, and *P. praecox* (Pomancillo Este and Santa Cruz) were located on the left side of the biplot and were characterized by high seed MC, and low seed mass, seed coat thickness, and seed width.

PC2 explained 32.4% of the variation and separated species and/or populations according to environmental variables and to seed coat thickness: seed width and seed coat thickness: seed mass ratios. All populations of *P. visco* and *G. decorticans* were associated with high altitude, latitude, and annual precipitation (AT), since they corresponded to positive scores on PC2. However, *P. praecox* (Recreo and Santa Cruz), *V. aroma* (Capital and Colonia del Valle), *S. mistol* (Huaycama and Recreo), and *S. lorentzii* (Santo Domingo), corresponding to negative scores, had high seed coat thickness: seed width and seed coat thickness: seed mass ratios and mean annual temperature (MAT). The remaining populations of *P. praecox*, *V. aroma*, *S. mistol*, and *S. lorentzii* had scores near zero on axis 1 and 2 and are characterized by a seed MC similar to that of *P. visco* and coat: width and coat: mass ratios similar to those of *G. decorticans.*

## 3. Discussion

### 3.1. Seed Traits and Seed Dormancy

The combination of morphological and physiological seed traits and environmental conditions (e.g., light, temperature, and water availability) result in the coordination of seed dispersal and germination at an optimal time for successful seedling establishment [8,39]. In our study, we found interspecific variability in all seed traits evaluated among the six native species growing in seasonally dry Chaco forests of Argentina. In general, the highest values for seed mass and morpho-anatomical seed traits were found in species (e.g., *G. decorticans*, *S. mistol*, and *S. lorentzii*) whose dispersal units were fruits, and the lowest in species whose dispersal units were seeds. All species showed differences in light and temperature requirements for germination. Thus, for *G. decorticans*, seed germination percentage was highest in darkness at a constant temperature, whereas *P. visco* seeds germinated to high percentages in all conditions evaluated. These characteristics would allow seeds of *G. decorticans* to germinate under buried conditions; whereas seeds of *P. visco* could germinate either under buried conditions or on the soil surface. For the other species, the low germination percentage in all light and temperature conditions indicates the presence of seed dormancy and the requirement for exposure of seeds to specific environmental conditions to promote dormancy-break and germination. Similar results have been reported for these species and other species characteristic of the seasonally dry Chaco forests of Argentina [38,40]. This interspecific variation in seed traits and seed germination could have important implications for the regeneration and coexistence (regeneration niche differentiation) of these species in the three kinds of Chaco seasonally dry forests inhabited by these species.

In our study, we confirmed our first hypothesis that species of Anacardiaceae, Fabaceae (except for *G. decorticans* that has ND seeds), and Rhamnaceae have seed dormancy (PY and/or PD). However, seeds of the legume *P. visco* are ND. Through initial water-imbibition tests and scanning electron microscope (SEM) analyses, we confirmed that seeds of *P. praecox* and *V. aroma* have PY with thick water-impermeable seed coats and a high thickness: seed mass ratio. The lens functions as water gap (Complex Type II simple water gap), which is characteristic of Fabaceae *sensu* Gama-Arachchige et al. [14]. For *P. visco*, the lack of PY was associated with a thin water-permeable seed coat and a low thickness: seed mass ratio. For *G. decorticans*, although the woody endocarp was thick, it was permeable to water, and seeds of *S. lorentzii* (Anacardiaceae) and *S. mistol* (Rhamnaceae) had PD, as confirmed by their low initial germination and the presence of a water-permeable seed coat.

It has been reported that seed mass, seed MC, and seed coat thickness are related to dormancy break [19]. These authors reported that seeds of small-seeded species tend to have a relatively low MC, thick seed coat, and PY; whereas those of large-seeded species tend to have a relatively high MC, thin seed coats, and be ND. In contrast to our second hypothesis, we found that seeds of large-seeded species have on average a low MC and high seed coat thickness and seed width, and they are ND (*G. decorticans*) or have PD (*S. mistol* and *S. lorentzii*). However, if we considered only Fabaceae species, seeds of *G. decorticans* and *P. visco* with high seed mass were ND, whereas those of *P. praecox* and *V. aroma* with low seed mass had PY. It has been suggested that species with large seeds have a proportionally thinner seed coat (i.e., reduced dormancy) than those with small seeds and that seedlings from large seeds have large reserves and can thrive in unfavorable environments better than those from small seeds with low reserves [12].

Similar to our results, other studies have shown that seeds of *P. praecox* and *V. aroma* have PY [28,29,32,33,34]. However, contrary to our results, seeds of *G. decorticans* [27], *S. mistol* [27,31], and *S. lorentzii* [32] had been reported to have PY. The difference between our results and those of other authors probably is due to differences in methods used to determine the class of dormancy. In previous studies for these three species, only methods to break PY were evaluated, and no imbibition studies were conducted, which are critical experiments to prove that seeds are water-impermeable [8]. The major problem with the methods in the previous studies is that they could have broken PD, which was confirmed in our study. Thus, for *S. mistol* and *S. lorentzii*, our results agree with the results of Baskin and Baskin [8,41] who mentioned that these species have PD. For *P. visco,* this is the first report of the absence of PY.

For *P. praecox*, mechanical and acid scarification and wet heat increased seed germination, and acid scarification and wet heat opened the water gap [29,30]. For *V. aroma*, only mechanical and acid scarification increased seed germination, and acid scarification opened the water gap [32,33]. For *S. mistol* and *S. lorentzii,* depending on the population, 3 months of dry storage significantly increased germination of intact seeds, suggesting that fresh seeds had non-deep PD that was broken via after-ripening during storage [41]. Interestingly, Venier et al. [33] reported that passage of *V. aroma* seeds through the digestive tract of cattle increased germination. Further, Renison et al. [27] reported that passage of *G. decorticans* and *S. mistol* seeds through the digestive tract of a large flightless bird native to South America increased germination.

**Figure 7:**
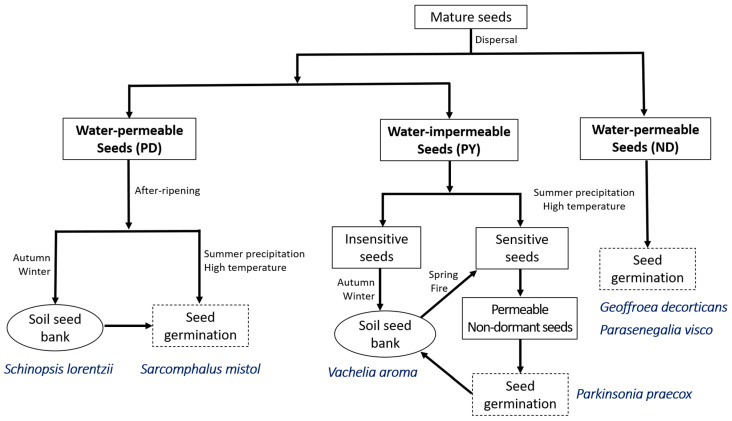
Model showing seed dormancy-break and germination of the six study species in the field. ND, nondormant; PD, physiological dormancy; and PY, physical dormancy.

Seeds of *G. decorticans* and *P. visco* (large-seeded species and ND) which are dispersed during the rainy season (summer) germinate immediately after dispersal, whereas those of *P. praecox* and *V. aroma* (small-seeded species and PY) are dispersed during the dry season (spring and autumn, respectively), and germinate in the following rainy season. During the dry season, seeds in the soil seed bank may be exposed to heat from spring fires that could open the water gap. These results suggest that the heat from fires can act as an environmental signal for the breaking of PY [42]. In addition, germination of *S. lorentizii* and *S. mistol* seeds is delayed until PD is broken via after-ripening, which can occur during the dry season (Figure 7). However, the role of environmental factors on the seed dormancy of these species requires further research.

Intraspecific variability of seed traits and seed dormancy and germination has been widely reported in many species and ecosystems around the world, and it has important consequences for the natural regeneration and persistence of species in their native habitats [8]. In our study, we also found high intraspecific variability in seed traits and seed dormancy and seed germination. Except for *P. praecox* and *S. lorentzii*, the study species exhibited intraspecific variability in most seed traits, probably due to a high phenotypic plasticity [12,38,43]. For seeds of *S. mistol* and *S. lorentzii*, (with PD), the response to dry storage differed among populations, which may be associated with differences in the intensity of PD and/or sensitivity to dormancy-breaking factors [18]. In contrast, seeds of *P. praecox* and *V. aroma* from all populations showed a similar pattern of response to dormancy-breaking methods, suggesting that seed dormancy is a conservative trait associated with phylogeny in these species [8]. Finally, intraspecific variation would give species a competitive advantage in the seasonal dry Chaco forests characterized by erratic precipitation and by temperatures that vary with latitude and altitude, and it may enhance colonization of species in new environments, particularly under scenarios of climate change [39].

### 3.2. Associations between Seed Traits, Seed Dormancy, and Environmental Variables

Geographical gradients of environmental factors such as temperature and precipitation offer opportunities for evaluating intra- and interspecific variability in plant traits and provide the possibility to analyze association between seed traits, seed dormancy, and environmental variables [44]. A positive correlation between dormancy and latitude and/or altitude has been widely reported [12,22,44,45]. In our study, we only found a negative correlation between the seed coat thickness: seed width and seed coat thickness: seed mass ratios and latitude, indicating that species and/or populations from high latitudes (southern population) had thicker seed coats in relation to seed width and mass than those from northern populations. However, southern species and/or populations have ND seeds. We also found that MC correlated negatively with seed mass and seed width, and coat thickness correlated positively with seed mass and seed width, contrary to the report by Rodrigues-Junior et al. [19] for Fabaceae. However, these results could be biased by the disproportionately higher values of seed mass, width, and coat thickness of *G. decorticans* compared with the other species.

Finally, species and populations were grouped in the PC Analysis according to seed mass, MC, seed width, and coat thickness; i.e., seed traits were species-specific. However, species and populations also were grouped according to environments variables, which partially supported our third hypothesis. That is, in all three Chaco vegetation units we found species with PY, PD, and ND. However, species and populations from sites with (on average) a higher altitude, latitude, and annual precipitation have ND seeds; whereas those from sites with (on average) a higher annual temperature have seeds with PY or PD. These results support the conclusion of Baskin and Baskin [13] that species with seed dormancy (PD and PY) are prevalent in seasonally dry environments. In the same sense, seed dormancy of *Pisum sativum* [44] and *Medicago trunculata* [12] is associated with increased annual temperatures. However, for other Fabaceae species, including *V. aroma*, other studies did not find a significant relationship between dormancy and rainfall or between dormancy and temperature gradients [34,46]. For *V. aroma*, Ferreras et al. [34] observed a trend toward a higher percentage of seed imbibition (lower PY) at the most humid extreme of a precipitation gradient in dry forests of the central region of Argentina.

Knowledge of seed traits and seed dormancy is essential for biodiversity conservation and can inform decisions for in situ and ex situ conservation and restoration management of species. Additionally, the information is needed for anticipating the response of species to current and/or future global climate changes [39]. Thus, it is essential to develop standard protocols for seed dormancy-break and germination for seed management in seed banks that guarantee the availability of high-quality seeds for their use in these programs. Further, it is important to identify intraspecific variability in these traits before recommending the use of specific seed sources and if restoration can be made at the species level. In this work, we have generated basic information on seed traits and seed dormancy, and their intraspecific variability, for six native tree species of Chaco forests that can be used for the implementation of ex situ conservation and ecological restoration programs.

## 4. Conclusions

We documented intra- and interspecific variability in seed traits and seed dormancy and their interactions in six native tree species belonging to the Anacardiaceae, Fabaceae and Rhamnaceae families growing in the seasonally dry Chaco forests of northwestern Argentina. For Fabaceae, two species had ND seeds (*G. decorticans* and *P. visco*), whereas seeds of two species (*P. praecox* and *V. aroma*) had PY. Seeds of *S. mistol* (Rhamnaceae) and *S. lorentzii* (Anacardiaceae) had PD. Thus, seeds of *G. decorticans* and *P. visco* can germinate following dispersal if soil moisture is favorable. Germination of *P. praecox* and *V. aroma* seeds is delayed until environmental conditions in the habitat, which may include heat from fires, cause the water gap to open, after which the timing of germination depends on the onset of the rainy season. Germination of *S. lorentzii* and *S. mistol* seeds is delayed until PD is broken via after-ripening, which can occur during the dry season. Seed dormancy was associated with seed mass but not with MC. Seeds of large-seeded species (*G. decorticans*, *P. visco*, *S. mistol*, *S. lorentzii*) were ND or had PD, and those of small-seeded species (*P. praecox* and *V. aroma*) had PY. Seeds of Fabaceae with relatively high seed mass and thin seed coats were ND, whereas those with low seed mass and thick seed coats had PY. Mechanical and chemical scarification were the most effective methods to break PY, and dry storage was effective in breaking PD.

Species and populations were grouped according to seed mass, MC, seed width and coat thickness, and environmental variables (altitude, latitude, and annual precipitation). Species inhabiting moist habitats (higher AP) had ND, whereas those from dry habitats (higher MAT) had PY or PD. These results suggest that seed traits and seed dormancy are species-specific, and that intraspecific variation in seed traits is likely associated with high phenotypic plasticity of species in response to local environmental heterogeneity. These findings are relevant not only for predicting the current and future natural regeneration of these species under possible scenarios of climate change but also for implementing ex situ conservation, management, and restoration programs urgently required for the persistence of Chaco forests in Argentina.

## 5. Materials and Methods

### 5.1. Studied Species and Seed Collection

The study species were *Geoffroea decorticans* (Gillies ex Hook. & Arn.) Burkart (Fabaceae), *Parasenegalia visco* (Lorentz ex Griseb.) Seigler and Ebinger (Fabaceae), *Parkinsonia praecox* (Ruiz and Pav. ex Hook.) Hawkins (Fabaceae), *Sarcomphalus mistol* (Griseb.) Hauenschild (Rhamnaceae), *Schinopsis lorentzii* (Griseb.) Engl. (Anacardiaceae) and *Vachellia aroma* (Gillies ex Hook. & Arn.) Seigler & Ebinger (Fabaceae). Fruiting occurs during October–December for *G. decorticans*, February–April for *P. visco*, November–March for *P. praecox* and *S. mistol*, March–August for *S. lorentzii*, and February–June for *V. aroma. G. decorticans* and *S. mistol* produce fleshy fruits (drupes) dispersed by animals, and *S. lorentzii* produces dry indehiscent fruits (samaras). *P. visco* and *P. praecox* produce dehiscent pods, whereas *V. aroma* produces indehiscent pods dispersed by animals (Figure 1). *P. praecox* and *V. aroma* colonize degraded areas, and presence of *V. aroma* indicates that fire has occurred in the habitat [47].

During the natural seed dispersal period for each species and population, many mature fruits were collected from at least 10 individuals per species and locality (hereafter population; Table 3). Seeds were cleaned, and if not used in the initial studies they were stored in paper bags at 20 °C and ≤30% relative humidity for 3 months. For *G. decorticans, S. mistol*, and *S. lorentzii* all experiments were carried out with fruits (hereafter seeds).

### 5.2. Seed Traits

Immediately after seed collection for each species and population, we determined seed viability, seed moisture content (MC), seed mass, seed germination percentage, and morpho-anatomical seed traits. Seed viability was evaluated in 4 replicates of 25 seeds each using the tetrazolium chloride (TZ) staining technique [48]. MC (expressed on a fresh weight basis) was determined gravimetrically by drying 3 samples of 10 seeds each at 103 °C for 17 h [48]. Seed mass was determined by weighing 30 fresh seeds individually with a precision balance to 0.0001 mg accuracy (Denver Instrument APX-200). Initial seed germination was evaluated in 4 replicates of 25 seeds per species and population. Seeds were placed in 9 cm diameter Petri dishes on two sheets of filter paper moistened with 5 mL distilled water and incubated in a germination chamber at 25 °C or 30/20 °C under light (8 h light/16 h dark; 30 μmol m–2 s–1, 400–700 nm of cool white fluorescent light) or complete darkness (24 h dark). Darkness was achieved by wrapping dishes in aluminum foil, and germination was recorded once at the end of the experiment. Temperature regimes were selected based on average temperatures occurring in the field during the summer–autumn months, which is the natural germination season [38]. At the end of the germination tests, when no additional germination had occurred in light for 2 weeks, a cut test was conducted to determine the viability of the non-germinated seeds (soft or firm, i.e., dead and viable, respectively), and the total number of viable seeds per replicate was calculated on the basis of the total number of firm non-germinated seeds + the germinated seeds.

The final germination percentage was calculated based on the total number of viable seeds. The time-course cumulative germination curves obtained at different treatments were used to estimate the time required for completion of 50% germination of viable seeds (t_50_). For this purpose, we fitted the time-course cumulative germination data to the Gompertz equation: Y = C x exp (−exp (−B x (X − M))), where Y is the percent of germination, X is time in days, C is the maximum percent of germination reached by the seed batches (the asymptote of the curve), B is the maximum germination rate standardized by the maximum percent of germination, and M is the value of time, in days, corresponding to the inflection point of the curve (moment at which the maximum rate of germination is reached).

Morpho-anatomical seed traits were evaluated for each species and each population in 10 intact and 10 transversally sectioned seeds that were fixed in FAA for 48 h. After fixation, the seeds were dehydrated in ethanol, critical-point dried with CO_2_, coated with gold, and observed with a scanning electron microscope (SEM) (JEOL JSM-6480 LV). Digital photographs of the hilar–micropyle zone and of cross sections of the seed coat were taken to determine morpho-anatomical traits. Photographs were processed with ImageJ [49] to estimate palisade layer thickness, seed coat thickness, and seed width. The water gap complex was characterized following Gama-Arachchige et al. [14].

### 5.3. Seed Dormancy

#### 5.3.1. Imbibition Curve

For each species and population, 25 intact and 25 mechanically scarified (seed coat opposite to the micropyle was cut/nicked with a scalpel) seeds were weighed using a digital balance (0.0001 g precision). Seeds were then sown in Petri dishes on two sheets of filter paper moistened with distilled water in light at 25 °C. Seeds were removed from the dishes at 1–2 h intervals for the first 12 h and then every 24 h, blotted dry with filter paper, and reweighed. The weighing procedure was continued until the mass of the seeds remained constant or the mechanically scarified seeds germinated. Water uptake was calculated for intact and scarified seeds as (final weight − initial weight)/initial weight × 100 [48].

#### 5.3.2. Dormancy-Breaking Treatments

The effects of mechanical scarification, dry heat, wet heat, and chemical scarification on dormancy-break and seed germination were evaluated for fresh seeds of *P. praecox*, *V. aroma* (both had impermeable seed coats, see imbibition curve results), and for seeds of *S. mistol* and *S. lorentzii* (these species had permeable seed coats and low initial germination, see seed traits and imbibition curve results) stored dry for 3 months. The following treatments were tested: (1) mechanical scarification: seed coat opposite the micropyle was nicked with a scalpel; (2) dry heat treatments: seeds were placed in oven at 90 °C for 10, 20, and 30 min; (3) wet heat treatments: seeds were immersed in water at 60, 80, and 100 °C for 2 min; and (4) chemical scarification: seeds were immersed in concentrated sulfuric acid for 10, 20, and 30 min. The control was intact seeds that received no pretreatments. For all treatments, 4 replicates of 25 seeds were used, and after each treatment (or control) seeds were then sown in Petri dishes on 2 sheets of filter paper moistened with distilled water and incubated in light at 25 °C. The number of germinated seeds and t_50_ were determined as previously described. The effects of mechanical scarification, dry heat, wet heat, and chemical scarification on morpho-anatomical changes (e.g., the opening of the water gap by removal or fissure of the lens) in seeds also were evaluated. Ten seeds of each species were submitted to each treatment and then their morpho-anatomical traits were observed as previously described.

### 5.4. Statistical Analyses

General linear mixed models were used to analyze initial viability, moisture content, seed mass, and morpho-anatomical seed traits, and compare among species or among populations for each species. Variance heterogeneity was considered, and the same information criteria were used to select the best fitted model. The same type of analysis was used to assess the effect of the factors on t_50_. General linear mixed models for binomial distribution and logit link functions were used to explain variations in number of germinated seeds. As the number of viable seeds was not the same for all experimental units, this number was entered as a covariable. For all analyses, species, scarification, light, and temperature treatment factors were considered as fixed effects. The population factor was considered as fixed when the species were analyzed separately, but since the localities were not the same for each species, this factor was considered to have random effects nested in the species when between-species comparisons were made. In all cases, all factor interactions were included in the models. AIC and BIC information criteria were used to select the most parsimonious model and the residual deviance/degrees of freedom ratio was calculated to assess if the goodness of fit of the model was reasonable and if there was over-dispersion. Treatments with no variance (none or all of the seeds germinated in each replicate) were excluded from the analyses. The post hoc DGC test of multiple comparisons of means was used to locate the differences among factor levels or their combinations of means when the effect of the factor or their interaction was significant (*p* < 0.05). We explored the associations between seed traits and environmental variables (latitude, longitude, altitude, MAT, AP), evaluating the significance of the Pearson correlation coefficient. Seed lots (of each species x population) were ordered and possible associations between seed characters (MC, seed mass, CT: seed coat thickness, W: seed width, C:W: seed coat thickness: seed width ratio, and C:M: seed coat thickness: seed mass ratio) and the environmental variables were evaluated using principal component analysis (PCA) on standardized data.

## Figures and Tables

**Table 1:** Mean and (standard deviation) of moisture content (MC; % fresh weight basis), seed mass, germination (%) in light and darkness at 25 and 30/20 °C, and water uptake (% initial mass basis) for each species and population.

Species	Population	MC (%)	Seed Mass (mg)	Germination (%)				Water Uptake (%)	
				Light		Darkness		Intact	Scarified
				25 °C	30/20 °C	25 °C	30/20 °C		
*Geoffroea decorticans*	Chacabuco	5.4 (0.3) ^Ca^	1005.2 (355) ^Aa^	16 (9) ^Cc^	7 (8) ^Cb^	83 (17) ^Ca^	77 (32) ^Ca^	71.2 (30) ^Ba^	63.9 (12) ^Ba^
	Huaycama	5.7 (0.5) ^Ca^	909.3 (115) ^Aa^	12 (5) ^Bc^	74 (28) ^Bb^	78 (19) ^Ba^	61 (41) ^Ba^	76.5 (37) ^Ba^	63.3 (11) ^Ba^
	Pomancillo Oeste	6.3 (0.1) ^Ca^	700.9 (246) ^Ab^	36 (18) ^Ac^	52 (31) ^Ab^	98 (5) ^Aa^	100	85.7 (16) ^Aa^	80.4 (19) ^Aa^
*Parasenegalia visco*	Concepción	8.9 (0.2) ^Ab^	111.3 (18) ^Ca^	100	100	100	100	233.4 (32) ^Ba^	223.0 (54) ^Aa^
	La Puerta	9.5 (0.04) ^Aa^	99.0 (15) ^Cb^	100	99 (3)	100	100	195.6 (193) ^Ca^	252.6 (45) ^Aa^
	Los Túneles	9.4 (0.3) ^Aa^	101.8 (18) ^Cb^	100	99 (3)	100	100	276.4 (46) ^Aa^	232.1 (38) ^Ab^
*Parkinsonia praecox*	Pomancillo Este	8.7 (0.2) ^Aa^	46.5 (5) ^Da^	44 (6) ^Aa^	50 (20) ^Aa^	38 (8) ^Ab^	26 (7) ^Ab^	64.2 (77) ^Ab^	150.2 (17) ^Aa^
	Recreo	9.0 (0.2) ^Aa^	42.5 (7) ^Da^	35 (10) ^Aa^	42 (14) ^Aa^	32 (17) ^Ab^	34 (15) ^Ab^	56.8 (86) ^Ab^	150.3 (36) ^Aa^
	Santa Cruz	8.7 (0.1) ^Aa^	46.2 (9) ^Da^	47 (5) ^Aa^	46 (15) ^Aa^	34 (6) ^Ab^	24 (18) ^Ab^	46.6 (72) ^Ab^	144.1 (39) ^Aa^
*Sarcomphalus mistol*	Huaycama	8.4 (0.1) ^Ba^	234.4 (38) ^Ba^	48 (7) ^Aa^	48 (8) ^Aa^	13 (18) ^Ab^	12 (13) ^Ab^	28.5 (5) ^Aa^	29.1 (3) ^Aa^
	Pomancillo Oeste	7.6 (0.1) ^Bc^	233.7 (37) ^Ba^	4 (4) ^Ca^	2(3) ^Ca^	0	0	19.1 (3) ^Ba^	19.7 (9) ^Ba^
	Recreo	7.8 (0.05) ^Bb^	176.9 (31) ^Bb^	28 (18) ^Ba^	29 (18) ^Ba^	5 (6) ^Bb^	8 (7) ^Bb^	26.5 (3) ^Aa^	29.7 (6) ^Aa^
*Schinopsis lorentzii*	El Portezuelo	8.6 (0.1) ^Aa^	118.8 (24) ^Ca^	37 (26) ^Ba^	40 (33) ^Bb^	26 (13) ^Ba^	25 (24) ^Bb^	76.5 (17) ^Aa^	67.9 (11) ^Ab^
	La Quebrada	8.5 (0.1) ^Aa^	128.2 (30) ^Ca^	55 (13) ^Aa^	41 (9) ^Ab^	43 (7) ^Aa^	39 (33) ^Ab^	78.2 (23) ^Aa^	77.6 (18) ^Aa^
	Santo Domingo	8.7 (0.1) ^Aa^	117.1 (27) ^Ca^	24 (13) ^Ca^	10 (4) ^Cb^	15 (15) ^Ca^	9 (4) ^Cb^	61.4 (9) ^Ba^	49.6 (9) ^Bb^
*Vachellia*	Capital	8.8 (0.1) ^Aa^	61.2 (7) ^Da^	8 (1) ^Ba^	4 (5) ^Ba^	8 (7) ^Ba^	4 (5) ^Ba^	18.0 (47) ^Ba^	152.8 (20) ^Ab^
*aroma*	Colonia del Valle	8.2 (0.2) ^Ab^	83.6 (10) ^Db^	10 (3) ^Ba^	9 (3) ^Ba^	8 (7) ^Ba^	5 (4) ^Ba^	2.9 (5) ^Ba^	167.0 (49) ^Ab^
	Pomancillo Oeste	9.3 (0.4) ^Aa^	66.7 (17) ^Da^	14 (6) ^Aa^	14 (4) ^Aa^	15 (18) ^Aa^	6 (8) ^Aa^	53.1 (82) ^Aa^	124.2 (22) ^Bb^

For seed MC and seed mass, values with different uppercase letters indicate significant differences between species and values with different lowercase letters indicate significant differences between populations of each species. For seed germination and water uptake, values with different uppercase letters indicate significant differences between populations and values with different lowercase letters indicate significant differences between treatments included in the analyses (DGC test *p* < 0.05).

**Table 2:** Mean and (standard deviation) of morpho-anatomical seed traits: palisade layer thickness (P), seed coat thickness (CT), seed width (W), coat thickness: seed width ratio (C:W) and coat thickness: seed mass ratio (C:M), for each species and population.

Species	Population	Pl (mm)	CT (mm)	W (mm)	C:W	C:M
*Geoffroea decorticans*	Chacabuco	-	4.21 (0.6) ^Aa^	14.69 (2.8) ^Aa^	0.29 (0.02) ^Ca^	0.004 (0.0003) ^Da^
	Huaycama	-	3.87 (0.1) ^Aa^	13.15 (0.6) ^Aa^	0.29 (0.01) ^Ca^	0.004 (0.001) ^Da^
	Pomancillo Oeste	-	2.49 (0.5) ^Ab^	10.09 (1.5) ^Aa^	0.24 (0.01) ^Cb^	0.003 (0.001) ^Da^
*Parasenegalia visco*	Concepción	0.036 (0.01) ^Ca^	0.14 (0.02) ^Fa^	1.54 (0.3) ^Da^	0.09 (0.01) ^Eb^	0.001(0.0003) ^Ea^
	La Puerta	0.029 (0.005) ^Ca^	0.11 (0.03) ^Fa^	1.46 (0.3) ^Da^	0.08 (0.01) ^Eb^	0.001 (0.0003) ^Ea^
	Los Túneles	0.030 (0.004) ^Ca^	0.14 (0.02) ^Fa^	1.19 (0.1) ^Da^	0.12 (0.03) ^Ea^	0.001 (0.0002) ^Ea^
*Parkinsonia praecox*	Pomancillo Este	0.063 (0.005) ^Ba^	0.32 (0.04) ^Ea^	1.47 (0.3) ^Da^	0.23 (0.05) ^Cb^	0.007 (0.001) ^Ba^
	Recreo	0.061 (0.003) ^Ba^	0.34 (0.05) ^Ea^	1.25 (0.2) ^Da^	0.28 (0.02) ^Ca^	0.008 (0.002) ^Ba^
	Santa Cruz	0.063 (0.01) ^Ba^	0.40 (0.07) ^Ea^	1.48 (0.2) ^Da^	0.27 (0.01) ^Ca^	0.009 (0.002) ^Ba^
*Sarcomphalus mistol*	Huaycama	-	1.15 (0.03) ^Ca^	3.72 (0.05) ^Ba^	0.31 (0.01) ^Bb^	0.005 (0.001) ^Ca^
	Pomancillo Oeste	-	1.13 (0.1) ^Ca^	3.32 (0.3) ^Ba^	0.34 (0.02) ^Ba^	0.004 (0.001) ^Ca^
	Recreo	-	1.29 (0.009) ^Ca^	3.62 (0.1) ^Ba^	0.36 (0.01) ^Ba^	0.007 (0.0003) ^Ca^
*Schinopsis lorentzii*	El Portezuelo	-	1.59 (0.4) ^Ba^	3.99 (0.9) ^Ba^	0.40 (0.03) ^Aa^	0.014 (0.004) ^Aa^
	La Quebrada	-	1.4 (0.3) ^Ba^	3.54 (0.6) ^Ba^	0.40 (0.02) ^Aa^	0.010 (0.003) ^Aa^
	Santo Domingo	-	1.46 (0.2) ^Ba^	3.52 (0.5) ^Ba^	0.42 (0.03) ^Aa^	0.013 (0.003) ^Aa^
*Vachellia aroma*	Capital	0.153 (0.03) ^Aa^	0.47 (0.1) ^Da^	2.62 (0.4) ^Ca^	0.18 (0.03) ^Da^	0.008 (0.003) ^Ba^
	Colonia del Valle	0.097 (0.02) ^Ab^	0.44 (0.09) ^Da^	2.55 (0.4) ^Ca^	0.17 (0.05) ^Da^	0.004 (0.0005) ^Ba^
	Pomancillo Oeste	0.106 (0.01) ^Ab^	0.46 (0.04) ^Da^	2.77 (0.4) ^Ca^	0.17 (0.01) ^Da^	0.007 (0.002) ^Ba^

Values with different uppercase letters indicate significant differences between species and values with different lowercase letters indicate significant differences between populations of each species (DGC test *p* < 0.05).

**Table 3:** Vegetation units and environmental variables (latitude; longitude; altitude; MAT, mean annual temperature; and AP, annual precipitation) for each species and population. Climatic data were extracted from WorldClim (https://www.worldclim.org/data/worldclim21.html; accessed on 1 November 2022). Population names correspond to the localities where seeds were collected in Catamarca Province, Argentina.

Species (Family)	Population	Vegetation Unit	Latitude	Longitude	Altitude (m a.s.l.)	MAT (°C)	AP (mm)
*Geoffroea decorticans* (Fabaceae)	Chacabuco	Arid Chaco	−28°26′	−65°43′	525	20.1	529
	Huaycama	Arid Chaco	−28°30′	−65°39′	578	19.0	529
	Pomancillo Oeste	Chaco Serrano	−28°18′	−65°43′	648	19.4	545
*Parasenegalia visco* (Fabaceae)	Concepción	Chaco Serrano	−28°40′	−66°04′	881	18.7	441
	La Puerta	Chaco Serrano	−28°11′	−65°46′	880	18.2	547
	Los Túneles	Chaco Serrano	−28°08′	−65°38′	955	18.3	613
*Parkinsonia praecox* (Fabaceae)	Pomancillo Este	Chaco Serrano	−28°18′	−65°42′	677	19.4	545
	Recreo	Semiarid Chaco	−29°05′	−65°4′	243	20.5	491
	Santa Cruz	Arid Chaco	−28°29′	−65°40′	566	20.1	530
*Sarcomphalus mistol* (Rhamnaceae)	Huaycama	Arid Chaco	−28°31′	−65°41′	538	20.1	524
	Pomancillo Oeste	Chaco Serrano	−28°18′	−65°43′	646	19.4	545
	Recreo	Semiarid Chaco	−29°08′	−65°05′	235	20.6	483
*Schinopsis lorentzii* (Anacardiaceae)	El Portezuelo	Chaco Serrano	−28°27′	−65°36′	760	18.5	553
	La Quebrada	Chaco Serrano	−28°27′	−65°51′	887	19.2	502
	Santo Domingo	Semiarid Chaco	−29°09′	−65°12′	254	20.5	483
*Vachellia aroma* (Fabaceae)	Capital	Arid Chaco	−28°26′	−65°45′	539	20.1	529
	Colonia del Valle	Arid Chaco	−28°38′	−65°54′	476	20.2	494
	Pomancillo Oeste	Chaco Serrano	−28°18′	−65°43′	653	19.4	545

## Data Availability

The data presented in this study are available on request from the corresponding author.
